# Global Biomonitoring of Perfluorinated Organics

**DOI:** 10.1100/tsw.2001.342

**Published:** 2001-11-06

**Authors:** John P. Giesy, Kurunthachalam Kannan, Paul D. Jones

**Affiliations:** National Food Safety and Toxicology Center, Department of Zoology, Michigan State University, East Lansing 48824, USA

**Keywords:** monitoring, perfluorinated compounds, PFOS, fluorochemical surfactants

## Abstract

The environmental distribution of fluorinated organic compounds (FOCs) has been less well described than the other halogenated hydrocarbons such as chlorinated and brominated compounds. This is despite the fact that FOCs have been used in a wide variety of products and applications for more than 50 years. FOCs are resistant to hydrolysis, photolysis, microbial degradation, or metabolism by vertebrates due to the high energy of carbon–fluorine bond. In particular, perfluorinated (fully fluorinated) compounds (PFCs) have the potential to persist in the environment. But, until recently, the extent and magnitude of environmental distribution of PFCs was unknown. Recent development of an analytical technique for PFCs using high performance liquid chromatography-negative ion electrospray tandem mass spectrometry (HPLC-ESMSMS)[1] permitted the survey of PFCs in livers and blood plasma of wildlife on a global scale[2].

Evidence for the presence of organic forms of fluorine in human blood was presented as early as 1968[3]. It was speculated that perfluorooctanoic acid (PFOA) and other synthetic fluorochemicals were the sources of PFCs found in the sera of general population. Only in the 1990s, using HPLC-ESMSMS analysis, were perfluorooctane sulfonate (PFOS) and PFOA identified in the blood sera of employees in the fluorochemical manufacturing industry at concentrations of up to 12.8 and 114 µg/mL, respectively[4]. The HPLC-ESMSMS analysis also detected perfluorooctanesulfonamide (PFOSA), PFOA and perfluorohexane sulfonate (PFHS) in human blood[1]. These studies raised questions regarding the magnitude and extent of wildlife exposures to PFCs since, prior to this study, neither it was known if exposures were regional or global nor if wildlife were exposed to PFCs. To address these questions, archived tissues of marine mammals, birds, fishes, reptiles, and amphibians from various parts of the world including Arctic and the Antarctic Oceans were analyzed for PFCs. About 1,700 samples have been analyzed since April 1999 to investigate the extent and magnitude of the distribution of PFCs in the global environment (Table 1). The study was focused on four perfluorinated compounds: PFOS, PFOSA, PFHS, and PFOA (Fig. 1).

Among the four PFCs monitored, PFOS was the most prevalent in the tissues of wildlife (Table 1). PFOSA, PFOA, and PFHS were detected in the tissues of a few species (<20% of the samples analyzed), generally at concentrations (approximately tenfold) less than those of PFOS. While PFOS is a metabolic product of various sulfonated perfluorochemicals, PFOSA, PFOA, and PFHS are intermediates in the production of several perfluorinated compounds. PFOSA and PFOA are also products used in various applications. PFOS was detectable in most samples including those from remote marine regions at concentrations greater than 1 ng/g[2,5,6].

**TABLE 1**
**Frequency and Maximum Concentrations of Detection of PFOS in Animal Tissues**

| Animal Class | n | Detection Frequency (%) | Maximum Conc (ng/g, wet) |
|---|---|---|---|
| Marine mammals | 400 | 77 | 1520 (liver) |
| Inland aquatic mammals (mink) | 200 | 100 | 4900 (liver) |
| Birds including penguins | 460 | 60 | 2220 (plasma) |
| Fishes and shellfish | 470 | 38 | 920 (muscle) |
| Terrestrial wild mammals | 50 | 50 | 200 (liver) |
| Frogs and turtles | 40 | 14 | 700 (liver) |

*Note:* Detection limits varied from 1 to 35 ppb, wet wt.

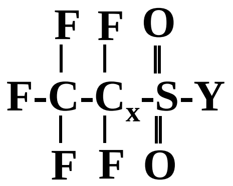

X= 4 to 10

Y = OH or $O^-M^+$ or $NH_2$

**FIGURE 1.** General Structure of perfluorinated sulfonates.

Concentrations of PFOS in the blood of ringed and grey seals from the Canadian and Norwegian Arctic ranged from 3 to 50 ng/mL. PFOS concentrations were two- to tenfold greater (14–230 ng/mL) in seals from relatively more contaminated locations such as the Baltic Sea. Similarly, blood plasma and serum of Laysan and black-footed albatrosses collected from remote oceanic locations such as Midway Atoll in the North Pacific Ocean contained PFOS at concentrations ranging from 3 to 26 ng/mL. PFOS concentrations in the blood of cormorants and herring gulls from the North American Great Lakes were approximately tenfold greater than those in albatrosses from Midway Atoll. While the liver of yellow-fin tuna from the northern North Pacific contained no detectable PFOS (<7 ng/g), blue-fin tuna from the Mediterranean Sea contained up to 87 ng/g PFOS in the liver. Similarly, livers of polar bear from Alaska contained concentrations of PFOS ranging from 180 to 680 ng/g, wet wt while PFOS concentrations in weddell seals from the Antarctic were less than the limit of quantification (35 ng/g). These results suggest that PFOS is distributed in remote areas including polar regions but that concentrations in wildlife from these areas were several-fold less than those from more industrialized and urbanized areas such as the Baltic Sea and the North American Great Lakes. Blood plasma of bald eagle fledglings collected from the midwestern U.S. contained PFOS concentrations up to 2570 ng/mL. Similarly, all fish eating bird tissues from Canada, Italy, Japan, and Korea contained detectable concentrations of PFOS. The occurrence of PFOS in these birds suggests that the potential source of exposure is the fish they consume. PFOS concentrations of up to 300 ng/g were detected in the muscle of carp from Saginaw Bay, Michigan. Among aquatic mammals, mink from the midwestern U.S. contained the greatest concentrations of PFOS (970–4900 ng/g liver, wet wt). Mink are opportunistic predators and a proportion of their diet is fish. Average concentrations of PFOS in Saginaw Bay carp, which were fed to mink, were 120 ng/g, wet wt, suggesting a biomagnification factor of 22 for PFOS in mink.

These results suggest that PFCs, particularly PFOS, are widely distributed on a global scale and can be persistent and bioaccumulative in the food chain. The mechanisms of transport and toxic effects of these compounds are the subjects of current investigations.

## References

1. Hansen, K.J., Clemen, L.A., Ellefson, M.E. and Johnson, H.O. (2001) Compound-specific, quantitative characterization of organic fluorochemicals in biological matrices. *Environ. Sci. Technol*. **35**, 766-770.
2. Giesy, J.P. and Kannan, K. (2001) Global distribution of perfluorooctane sulfonate and related perfluorinated compounds in wildlife. *Environ. Sci. Technol.* **35**, 1339-1342.
3. Taves, D.R. (1968) Evidence that there are two forms of fluoride in human serum. *Nature* **217**, 1050-1051.
4. Olsen, G.W., Burris, J.M., Burlew, M.M. and Mandel, J.H. (2000) Plasma cholecystokinin and hepatic enzymes, cholesterol and lipoproteins in ammonium perfluorooctanoate production workers. *Drug Chem. Toxicol*. **23**, 603-620.
5. Kannan, K., Hansen, S.P., Franson, C.J., Bowerman, W.W., Hansen, K.J., Jones, P.D. and Giesy, J.P. (2001) Perfluorooctane sulfonate in fish-eating water birds including bald eagles and albatrosses. *Environ. Sci. Technol*. **35,** 3065-3070.
6. Kannan, K., Koistinen, J., Beckmen, K., Evans, T., Gorzelany, J., Hansen, K.J., Jones, P.D. and Giesy, J.P. (2001) Accumulation of perfluorooctane sulfonate in marine mammals. *Environ. Sci. Technol*. **35**, 1593-1598.
